# Distinct genetic abnormalities in cerebrospinal fluid versus tumor tissue and plasma in a SCLC patient with leptomeningeal metastasis: A case report

**DOI:** 10.1097/MD.0000000000043283

**Published:** 2025-07-18

**Authors:** Xiaozhen Chen, Jianbo Zhu, Hongya Dai, Bo Zhu, Zhongyu Wang

**Affiliations:** a Institute of Cancer, Xinqiao Hospital, Third Military Medical University, Chongqing, People’s Republic of China; b Chongqing Key Laboratory of Immunotherapy, Chongqing, People’s Republic of China.

**Keywords:** CDK4 copy number increase, leptomeningeal metastasis, next-generation sequencing, small cell lung cancer

## Abstract

**Rationale:**

Leptomeningeal metastasis (LM) in small cell lung cancer is rare, and the application of next-generation sequencing in these cases remains limited.

**Patient concerns:**

This study presents a case of a small cell lung cancer patient who developed LM despite achieving a partial response in extracranial tumors. Notably, next-generation sequencing analysis of tumor tissue, blood, and cerebrospinal fluid (CSF) uniquely revealed an increased copy number of cyclin-dependent kinase 4 specifically in the CSF.

**Diagnoses:**

A lumbar puncture was performed, and pathological examination of the CSF confirmed small cell carcinoma.

**Interventions:**

The patient received intrathecal methotrexate therapy and was scheduled promptly for whole-brain radiation therapy.

**Outcomes:**

Unfortunately, the patient passed away at home 3 days after discharge.

**Lessons:**

This case underscores the importance of prompt diagnosis of small cell lung cancer with suspected LM through CSF cytology obtained via lumbar puncture. It also highlights the potential of CSF as a valuable liquid biopsy medium for tracking disease progression and treatment response in central nervous system malignancies, providing a basis for the development of targeted therapeutic strategies.

## 1. Introduction

Brain metastasis frequently occurs in patients with small cell lung cancer (SCLC); however, leptomeningeal metastasis (LM) is rare and infrequently reported. Compared to brain metastasis, LM is associated with poorer outcomes, typically resulting in survival times of only 2 to 4 months.^[1]^ While next-generation sequencing (NGS) of cerebrospinal fluid (CSF) is commonly utilized to track tumor evolution in driver gene-positive non-SCLC patients with LM,^[2]^ genetic mutations are less frequent in SCLC, making CSF-based NGS analysis uncommon and poorly documented. This case report is the first to investigate genetic differences among CSF, tumor tissue, and plasma using NGS in an SCLC patient with LM, aiming to shed light on the mechanisms underlying LM and guide precision therapy strategies.

## 2. Case report

**Figure 1.**
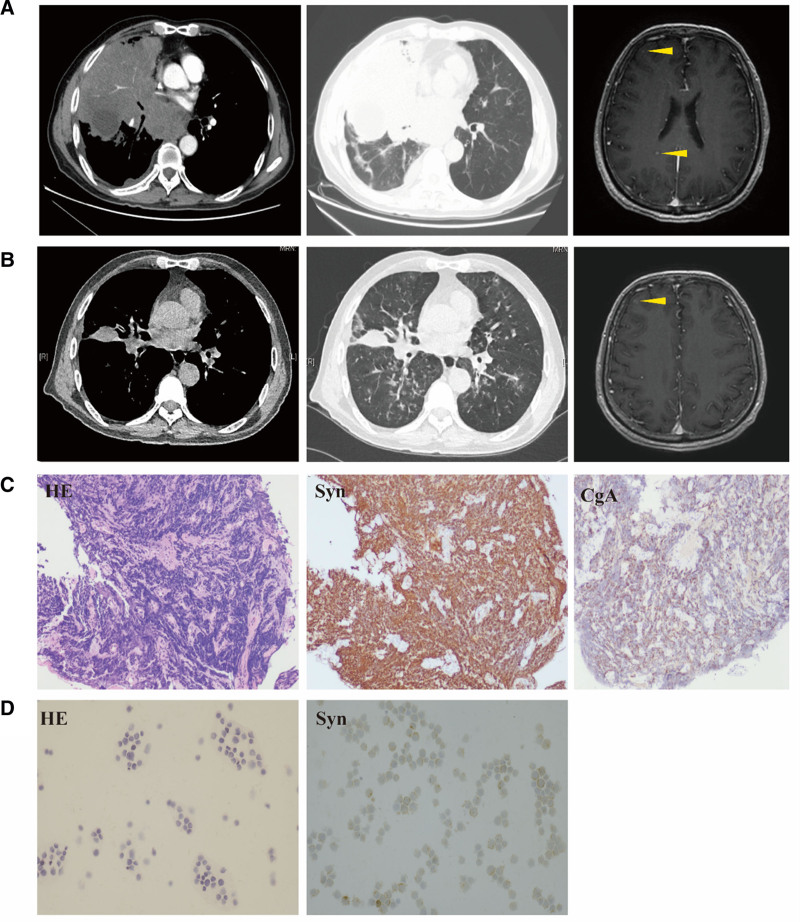
Radiological and pathological assessments of the patient. (A) Baseline chest computed tomography (CT) demonstrated a large mass in the right lung hilum and mediastinum with accompanying obstructive atelectasis (left and middle panels). Cranial magnetic resonance imaging (MRI) identified metastases in the right frontal lobe and the posterior horn of the lateral ventricle (right panel). (B) After therapy, a partial response was observed (left and middle). The nodules in the right frontal lobe have increased in size compared to the previous scan, while the nodules in the posterior horn of the lateral ventricle have disappeared (right). (C) At the time of initial diagnosis, hematoxylin and eosin (H&E) staining of the bronchoscopic biopsy specimen demonstrated small round cells (left), while immunohistochemical analysis showed positive staining for synaptophysin (middle) and chromogranin A (right). (D) H&E staining of the cerebrospinal fluid (CSF) demonstrated small cell carcinoma cells (left), with immunohistochemical analysis confirming positivity for synaptophysin (right). CSF = cerebrospinal fluid, CT = computed tomography, H&E = hematoxylin and eosin, MRI = magnetic resonance imaging.

A 59-year-old Chinese male, with a 40-year smoking history, presented to the hospital complaining of coughing, productive sputum, and hemoptysis persisting for 1 year. Chest and abdominal computed tomography scans identified a substantial mass located in the right lung hilum and mediastinum, with associated obstructive atelectasis (Fig. 1A, left). The scans further revealed invasion into the right pulmonary vessels, accompanied by tumor thrombus formation, as well as involvement of the pericardium and pleura. Extensive metastatic lesions were observed in lymph nodes at multiple sites, including bilateral cervical roots, mediastinum, right hilum, right cardiophrenic angle, and hepatogastric space. Further imaging confirmed metastases in the right adrenal gland and liver. Magnetic resonance imaging (MRI) of the brain identified metastatic lesions in the right frontal lobe and posterior horn of the lateral ventricle (Fig. 1A, right), while bone scanning demonstrated metastasis to the left iliac crest. Histological examination via bronchoscopic biopsy from the right middle lobe confirmed SCLC, with immunohistochemistry showing TIF(−), Ki-67 (approximately 95%), Syn(+), and CgA(+); Fig. 1C). The patient was ultimately diagnosed with extensive-stage SCLC.

The patient subsequently received a programmed cell death protein 1 inhibitor combined with chemotherapy based on the etoposide and carboplatin regimen. After completing 4 cycles of this combined therapy, computed tomography imaging indicated a partial tumor response according to the response evaluation criteria in solid tumors version 1.1 criteria (Fig. 1B, left). After the fifth cycle, the patient experienced dizziness, progressively worsening headaches, and bilateral weakness in the lower limbs. MRI of the brain showed a slight enlargement of the lesion in the right frontal region compared to previous scans (Fig. 1B, right). However, given the small size of the parenchymal lesions and the disproportionate severity of clinical symptoms relative to MRI findings, LM was suspected. A lumbar puncture was performed, and pathological examination of the CSF confirmed small cell carcinoma (Fig. 1D). The patient received intrathecal methotrexate therapy and was scheduled promptly for whole-brain radiation therapy. During radiation treatment, the patient’s headaches intensified, and a pulmonary infection developed. Subsequently, the patient and family declined further treatment, leading to hospital discharge. Unfortunately, the patient passed away at home 3 days after discharge.

**Figure 2.**
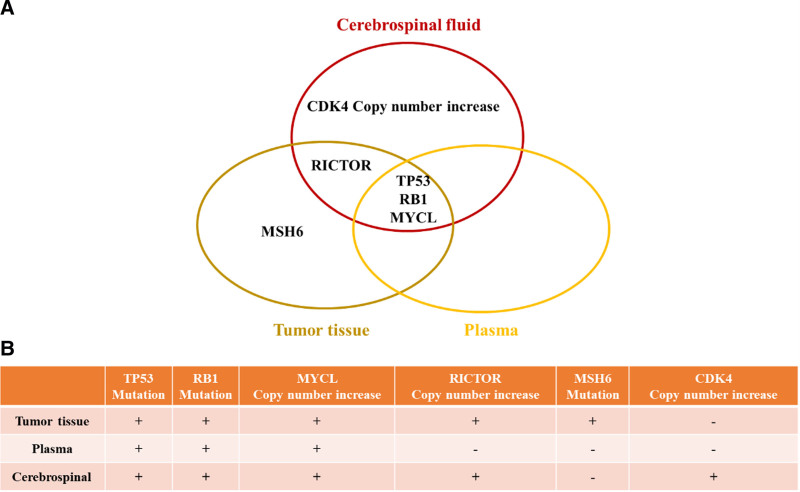
(A and B) Comparison of genetic alterations in tumor tissue, plasma, and cerebrospinal fluid as identified by next-generation sequencing, illustrating differences in mutational landscapes among the 3 sample types. CDK4 = cyclin-dependent kinase 4, MSH6 = MutS homolog 6, MYCL = MYC proto-oncogene, BHLH transcription factor like, RB1 = retinoblastoma 1, RICTOR = RPTOR independent companion of MTOR complex 2, TP53 = tumor protein P53.

NGS of the patient’s tumor tissue, blood, and CSF identified mutations in tumor protein P53, retinoblastoma 1, and MYC proto-oncogene, BHLH transcription factor like across all 3 specimens. In contrast, RPTOR independent companion of MTOR complex 2 copy number amplification was detected in both the tumor tissue and CSF but was absent in plasma. A mutation in MutS homolog 6 was found solely in the tumor tissue. Interestingly, cyclin-dependent kinase 4 (CDK4) copy number amplification was identified exclusively in the CSF (Fig. 2).

## 3. Discussion

In this case, the SCLC patient experienced rapid progression of LM during a partial response in extracranial lesions following chemotherapy combined with immunotherapy. This progression may be attributed to the limited ability of therapeutic agents to cross the blood-brain barrier and achieve effective concentrations in the central nervous system (CNS).^[3]^ Although immune responses were detected in the CSF, immune checkpoint inhibitors have the potential to act on both primary tumors and meningeal metastases, leading to reduced lesion burden and improvements in neurological and cognitive symptoms.^[4]^ The aggressive progression of LM in this patient may be associated with specific genetic alterations in the CSF, which could have fostered an immunosuppressive microenvironment and disrupted inflammatory signaling pathways, thereby contributing to immune resistance.^[5]^

NGS results indicate revealed notable differences in genetic alterations among CSF, tumor tissue, and plasma. Mutations in tumor protein P53 and retinoblastoma 1, along with MYC proto-oncogene, BHLH transcription factor like amplifications, which are key drivers well-established in the pathogenesis of SCLC were consistently detected across all 3 sample types.^[6]^ In contrast, RPTOR independent companion of MTOR complex 2 copy number amplification was identified in tumor tissue and CSF but absent in plasma, possibly indicating a lower circulating tumor burden or a dilution effect within the bloodstream. The exclusive presence of the MutS homolog 6 mutation in tumor tissue highlights the limitations of liquid biopsy in capturing certain genomic alterations and points to a localized mutation confined to the primary tumor microenvironment.^[7]^ Notably, the detection of CDK4 copy number amplification solely in the CSF may represent a distinct mechanism of tumor spread to the CNS. This observation is consistent with previous findings linking CDK4 amplification to aggressive tumor phenotypes and CNS involvement.^[8]^ Moreover, given the limited therapeutic options for SCLC, CDK4 inhibitors may offer a promising targeted approach for patients with such alterations. These discrepancies likely reflect the unique microenvironment of the CSF, where the blood-brain barrier restricts drug penetration, potentially allowing specific cancer subclones to evade treatment and proliferate. Moreover, the CNS may serve as a sanctuary site where preexisting subclones with particular genetic mutations preferentially accumulate.^[9]^

In conclusion, this case underscores the importance of prompt diagnosis of SCLC with suspected LM through CSF cytology obtained via lumbar puncture. It also highlights the potential of CSF as a valuable liquid biopsy medium for tracking disease progression and treatment response in CNS malignancies, providing a basis for the development of targeted therapeutic strategies.

## Acknowledgments

The authors would like to thank the MJEditor (www.mjeditor.com) for providing English editing services during the preparation of this manuscript.

## Author contributions

**Conceptualization:** Xiaozhen Chen, Bo Zhu.

**Data curation:** Xiaozhen Chen, Jianbo Zhu, Hongya Dai.

**Formal analysis:** Xiaozhen Chen.

**Funding acquisition:** Zhongyu Wang.

**Methodology:** Jianbo Zhu.

**Software:** Hongya Dai.

**Supervision:** Bo Zhu, Zhongyu Wang.

**Writing** – **original draft:** Xiaozhen Chen.

**Writing** – **review & editing:** Zhongyu Wang.

## References

[1] Thakkar JP Kumthekar P Dixit KS Stupp R Lukas RV. Leptomeningeal metastasis from solid tumors. J Neurol Sci. 2020;411:116706. 32007755 10.1016/j.jns.2020.116706

[2] Pentsova EI Shah RH Tang JB. Evaluating cancer of the central nervous system through next-generation sequencing of cerebrospinal fluid. J Clin Oncol. 2016;34:2404–15. 27161972 10.1200/JCO.2016.66.6487 PMC4981784

[3] Berghoff AS Preusser M. Role of the blood-brain barrier in metastatic disease of the central nervous system. Handb Clin Neurol. 2018;149:57–66. 29307361 10.1016/B978-0-12-811161-1.00004-9

[4] Brastianos PK Lee EQ Cohen JV. Publisher Correction: Single-arm, open-label phase 2 trial of pembrolizumab in patients with leptomeningeal carcinomatosis. Nat Med. 2020;26:1309. 10.1038/s41591-020-0978-1 32555425

[5] Rios-Hoyo A Arriola E. Immunotherapy and brain metastasis in lung cancer: connecting bench side science to the clinic. Front Immunol. 2023;14:1221097. 37876939 10.3389/fimmu.2023.1221097 PMC10590916

[6] George J Lim JS Jang SJ. Comprehensive genomic profiles of small cell lung cancer. Nature. 2015;524:47–53. 26168399 10.1038/nature14664 PMC4861069

[7] Nikanjam M Kato S Kurzrock R. Liquid biopsy: current technology and clinical applications. J Hematol Oncol. 2022;15:131. 36096847 10.1186/s13045-022-01351-y PMC9465933

[8] Yang B Luo LH Luo W. The genomic dynamics during progression of lung adenocarcinomas. J Hum Genet. 2017;62:783–8. 28381877 10.1038/jhg.2017.40 PMC5537414

[9] Brastianos PK Carter SL Santagata S. Genomic characterization of brain metastases reveals branched evolution and potential therapeutic targets. Cancer Discov. 2015;5:1164–77. 26410082 10.1158/2159-8290.CD-15-0369 PMC4916970

